# Application of bone alkaline phosphatase and 25-oxhydryl-vitamin D in diagnosis and prediction of osteoporotic vertebral compression fractures

**DOI:** 10.1186/s13018-023-04144-2

**Published:** 2023-09-30

**Authors:** Yuelin Chen, Xiaolin Sun, Xiaofei Sui, Yan Li, Zhen Wang

**Affiliations:** 1 Spinal Surgery, Zibo First Hospital, Zibo, 255200 Shandong China; 2 Clinical Laboratory, Zibo First Hospital, Zibo, 255200 Shandong China; 3 Orthopedics and Traumatology Department II, Penglai Traditional Chinese Medicine Hospital, Yantai, 265600 Shandong China; 4 Nursing, Penglai Traditional Chinese Medicine Hospital, Yantai, 265600 Shandong China; 5 Spinal Surgery, Tai’an Central Hospital Affiliated to Qingdao University, Taian, 271000 Shandong China

**Keywords:** Osteoporosis, Spinal fracture, Deep convolutional neural network, Computed tomography images, Bone alkaline phosphatase, 25-Oxhydryl-vitamin

## Abstract

**Background:**

Osteoporosis is a bone metabolic disease that usually causes fracture. The improvement of the clinical diagnostic efficiency of osteoporosis is of great significance for the prevention of fracture. The predictive and diagnostic values of bone alkaline phosphatase (B-ALP) and 25-oxhydryl-vitamin D (25-OH-VD) for osteoporotic vertebral compression fractures (OVCFs) were evaluated.

**Methods:**

110 OVCFs patients undergoing percutaneous vertebroplasty were included as subjects and their spinal computed tomography (CT) images were collected. After that, deep convolutional neural network model was employed for intelligent fracture recognition. Next, the patients were randomly enrolled into Ctrl group (65 cases receiving postoperative routine treatment) and VD2 group (65 cases injected with vitamin D2 into muscle after the surgery). In addition, 100 healthy people who participated in physical examination were included in Normal group. The differences in Oswestry dysfunction indexes (ODI), imaging parameters, B-ALP and 25-OH-VD expressions, and quality of life (QOL) scores of patients among the three groups were compared. The values of B-ALP and 25-OH-VD in predicting and diagnosing OVCFs and their correlation with bone density were analyzed.

**Results:**

It was demonstrated that computer intelligent medical image technique was more efficient in fracture CT recognition than artificial recognition. In contrast to those among patients in Normal group, B-ALP rose while 25-OH-VD declined among patients in Ctrl and VD2 groups (*P* < 0.05). Versus those among patients in Ctrl group, ODI, Cobb angle, and B-ALP reduced, while bone density, the height ratio of the injured vertebrae, 25-OH-VD, and QOL score increased among patients in VD2 group after the treatment (*P* < 0.05). The critical values, accuracy, and areas under the curve (AUC) of the diagnosis of OVCFs by B-ALP and 25-OH-VD amounted to 87.8 μg/L versus 30.3 nmol/L, 86.7% versus 83.3%, and 0.86 versus 0.82, respectively. B-ALP was apparently negatively correlated with bone density (r =  − 0.602, *P* < 0.05), while 25-OH-VD was remarkably positively correlated with bone density (r = 0.576, *P* < 0.05).

**Conclusion:**

To sum up, deep learning-based computer CT image intelligent detection technique could improve the diagnostic efficacy of fracture. B-ALP rose while 25-OH-VD declined among patients with OVCFs and OVCFs could be predicted and diagnosed based on B-ALP and 25-OH-VD. Postoperative intramuscular injection of VD2 could effectively improve the therapeutic effect on patients with OVCFs and QOL.

## Introduction

Osteoporosis is a bone metabolic disease. The reduction of bone density and bone mass among patients with osteoporosis is induced by multiple factors, which eventually causes damages to bone microstructures. In this case, the incidence of fracture is extremely high [1]. Osteoporosis-caused fracture usually occurs in vertebral column, wrist joint, hip joint, and humerus. The incidence of spinal fracture is high [2]. Vertebral column is a weight-bearing objects. Bone structure gradually becomes hollow among elderly people so that minor external force leads to fracture followed by osteoporotic vertebral compression fractures (OVCFs). OVCFs affects quality of life (QOL) and leads to long-term death in severe cases [3, 4]. Therefore, early diagnosis of OVCFs can effectively reduce the mortality of patients. In clinical practice, magnetic resonance imaging (MRI), computed tomography (CT), and ultrasound are often applied to the diagnosis of OVCFs [5]. CT images have the advantages of quick examination, high popularization rate, and high detection rate and quantitative CT images can be employed to detect bone density [6]. However, the diagnostic accuracy declines due to blurred images caused by respiratory artifacts during CT examination. With the rapid development of computer image analysis technology, safe medical image and computer intelligent detection are widely applied in medical field [7]. As to computer medical image intelligent analysis technology, the automatic detection, analysis, and recognition of medical images are realized. Deep convolutional neural network (DCNN) can be employed to classify and recognize images [8, 9]. Nonetheless, there are only a few studies on the application of computer image intelligent analysis technology in CT diagnosis of fracture.

Redlich et al. [10] showed that sclerotin status and the incidence of osteoporosis could be predicted based on the detection of bone density among premenopausal females with systemic lupus erythematosus by quantitative imaging technique combined with laboratory indicators. Quantitative CT combined with bone biochemical marker detection could improve the diagnostic reliability of OVCFs. Bone alkaline phosphatase (B-ALP) is the phenotypic marker for osteoblasts that directly reflects the activity and functional status of osteoblasts. Hence, it is applied to the evaluation of bone mineralization disorder or metabolic bone disease [11]. 25-oxhydryl-vitamin D (25-OH-VD) is the main form of vitamin D in human body and an essential component for maintaining health and cell growth and development [12]. Silva et al. [13] held that vitamin D deficiency led to osteomalacia and the marginal concentration of 25-OH-VD was associated with the occurrence of osteoporosis. Nonetheless, there are only a small number of studies on the correlation between B-ALP and 25-OH-VD and the effects of the prediction and diagnosis of OVCFs and between B-ALP and 25-OH-VD and bone density.

In the context of safe medical images, the application effect of computer intelligent technology on the analysis and recognition of fracture CT images was investigated. The predictive and diagnostic values of B-ALP and 25-OH-VD for OVCFs were evaluated. Besides, the effects of intramuscular injection of vitamin D2 after percutaneous vertebroplasty (PVP) on the improvement and prognosis of clinical symptoms were compared to provide some references for searching for early diagnostic markers of OVCFs, improving cure rate, and reduce mortality.

## Materials and methods

### General data

110 OVCFs patients treated in Tai’an Central Hospital affiliated to Qingdao between May, 2020 and December, 2021 and 100 healthy subjects were included.

The inclusion criteria were as follows.

A. Patients who met the diagnostic criteria for osteoporosis in Chinese Medical Association Guidelines for the Diagnosis and Treatment of Osteoporosis (2016 edition).
B. Patients diagnosed with OVCFs through CT imaging examination.
C. Patients with complete spinal vertebral body posterior wall and undamaged vertebral pedicle.
D. Patients without the signs of nerve root or spinal cord injury.
E. Patients who were tolerant of surgical treatment.

The exclusion criteria were as follows.

A. Patients with pathological fracture.
B. Patients with open injury or fracture and dislocation in other sites.
C. Patients with a history of old fracture.
D. Patients with heart, liver, and kidney dysfunctions.
E. Patients with bone metastatic tumors or other malignant tumors.
F. Patients who were unconscious and unable to communicate normally.
G. Female patients were examined for hormone levels and menstrual history to exclude menopausal patients.

Healthy subjects were included in the control group, while patients with osteoporotic vertebral compression fractures (OVCF) were randomly divided using a random number table method into a control group (55 cases) and a VD2 group (55 cases). The control group consisted of 27 males and 28 females, with ages ranging from 46 to 64 years. The average age was 55.38 ± 6.47 years. The disease duration ranged from 1 to 8 days, with an average of 5.16 ± 1.34 days. The body mass index (BMI) ranged from 18 to 25 kg/m^2^, with an average BMI of 22.08 ± 1.25 kg/m^2^. The VD2 group comprised 29 males and 26 females, aged between 46 and 65 years, with an average age of 56.31 ± 6.81 years. The disease duration ranged from 1 to 9 days, with an average of 5.20 ± 1.46 days. The BMI ranged from 18 to 24 kg/m^2^, with an average BMI of 22.42 ± 1.38 kg/m^2^. General data differences among the three groups of patients were not statistically significant (*P* > 0.05). The implementation of this study was approved by the Ethics Committee of Qingdao Municipal Affiliated Taian Central Hospital, and the enrolled subjects have signed informed consent forms.

### Computer CT image intelligent analysis

**Fig. 1:**
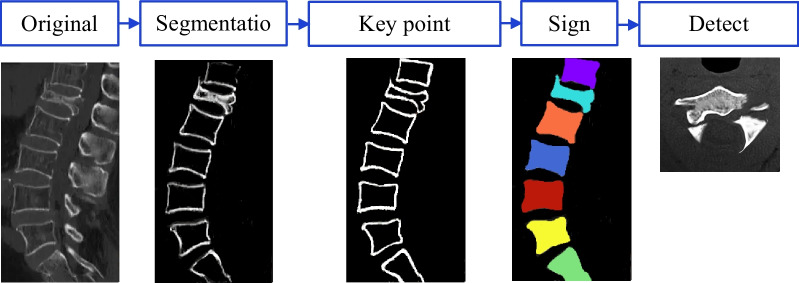
CT computer intelligent segmentation process

A 64-slice spiral CT imaging system was utilized to scan thoracic vertebrae or lumbar vertebrae of included patients. Patients were instructed to take supine position for CT scan. The scan parameters were set as follows. Tube voltage, tube current, pitch, collimation width, slice thickness, and overlap were set as 120 kV, 160 mAs, 0.935, 64 × 0.5 mm^2^, 0.63 mm, and 60%, respectively. The pixel values of the obtained CT images were normalized and then flipped horizontally, rotated counterclockwise 15°, rotated clockwise 15°, and cropped to expand image data. Besides, DCNN was constructed for the analysis of CT images. The model included segmentation, key point detection, and fracture detection networks. The detection process was presented in Fig. 1 below.

Firstly, the improved V-Net model was employed to segment osseous tissues in CT images. In addition, Dice coefficient was utilized to evaluate the segmentation effect. The calculation method for Dice coefficient was displayed as Eq. (1).

1 $$Dice=\frac{2\left|A\cap B\right|}{\left|A\right|+\left|B\right|}$$

In Eq. (1), $\left|A\cap B\right|$, $\left|A\right|$, and $\left|B\right|$ represented the intersection of data sets A and B, the number of elements in data set A, and the number of elements in data set B, respectively.

Then, virtual resource block (VRB) network was used to detect the key points. Dilation algorithm was adopted to inflate the key points into regions and determine whether output outcomes of the model were in key regions. Finally, vertebrae were marked and fracture sites were recognized. The differences between the output outcomes and gold standard overlap regions were evaluated. Dice > 0.8 was set as the standard for determining fracture recognition result. What’s more, precision, recall, and F1 were utilized to evaluate fracture recognition effect of computer medical image analysis model. The calculation methods for various indicators were shown as Eqs. (2), (3), and (4).

2 $$Precision=\frac{TP}{TP+FP}$$

3 $$Recall=\frac{TP}{TP+FN}$$

4 $$F1=\frac{2TP}{2TP+FP+FN}$$

In Eqs. (2), (3), and (4), TP, FP, and FN referred to the number of positive samples predicted to be positive, negative samples predicted to be positive, and positive samples predicted to be negative, respectively.

### Treatment methods

The patients in Ctrl and VD2 groups underwent PVP. They were instructed to take prone position for the establishment of vein passage. After the routine vital sign monitor was connected, local anesthesia was performed and lateral vertebral arch puncture was performed along the circular cortical outer edge of vertebral pedicle. During wire drawing, polymethyl methacrylate bone cement was slowly injected and the puncture needle was removed after bone cement was solidified. Next, routine hemostasis, suture, and anti-infection were carried out. After the surgery, patients in Ctrl group orally took calcium carbonate D3 tablets (manufacturer: Wyeth Pharmaceutical Co., Ltd.; Product specification: 600 mg*60 tablets; approval number: SFDA H10950029). The dose was 1 tablet per day. Based on the therapy for patients in Ctrl group, the patients in VD2 groups were injected with vitamin D2 continuously through muscle after the surgery (manufacturer: Kivipharm; product specification: 1 mL:10 mg 400,000 units; approval number: SFDA H13022468). The injection dose was 400,000 U/d and vitamin D2 was injected for 7 consecutive days. After that, X-ray films were reviewed to observe the growth of callus. In addition, patients were ordered to perform functional exercise.

### Observation indexes

① Oswestry dysfunction index (ODI) scale [14] was utilized to evaluate the level of dysfunction 3 months after the treatment. ODI scale included pain intensity, standing, sitting, walking, holding, sleep, travel, life, social contact, and sexual life. 6-point scoring method was adopted. A higher ODI suggested severer dysfunction after the treatment.
② After the treatment, X-ray and CT imaging technique were used to evaluate therapeutic effects. Lumbar spine anteroposterior projection was implemented. In addition, the ratio of the height of fractured lumbar vertebra to that of adjacent normal lumbar vertebra and Cobb angle were measured. Quantitative CT was utilized to measure bone density.
③ 2 mL venous blood were collected in fasting state before and after the treatment. Fully automatic electrochemi- luminescence immunoassay instrument was employed to detect the expressions of specific B-ALP and 25-OH-VD.
④ QOL scale [15] was utilized to evaluate QOL of patients 3 months after the treatment. QOL scale included pain, somatic function, vitality, emotional role, physical role, mental health, and social function. 4-point scoring method was adopted. A higher QOL score indicated lower QOL after the treatment.

### Statistical processing

SPSS19.0 was utilized for statistical analysis. Measurement data were denoted by $\left(\overline{x }\pm s\right)$ and the differences between groups were compared using t test. Enumeration data were expressed as percentage and the differences between groups were compared using *χ*^2^ test. Receiver operating characteristic curves (ROCs) were drawn to evaluate the diagnostic values of B-ALP and 25-OH-VD for OVCFs. Pearson test was employed to analyze the correlation between B-ALP as well as 25-OH-VD and bone density. *P* < 0.05 suggested the differences between groups revealed statistical significance.

## Results

### Effects of computer CT image intelligent detection

**Fig. 2:**
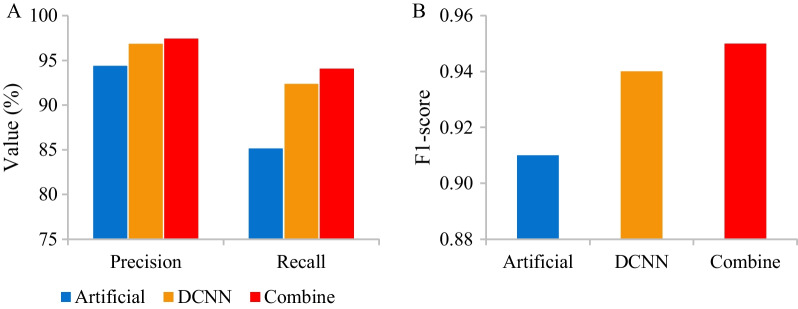
Evaluation of the effectiveness of computer CT intelligent detection. **A** Precision and recall. **B** F1 scores

The differences in the precision, recall, and F1 of the detection of OVCFs by artificial detection, DCNN model, and artificial detection combined with DCNN model were analyzed and compared. Precision, recall, and F1 of the above detection methods amounted to 94.36% versus 96.83% versus 97.42%, 85.12% versus 92.35% versus 94.06%, and 0.91 versus 0.94 versus 0.95. Precision, recall, and F1 of the combined detection were the highest followed by those of DCNN. The effect of artificial detection was the poorest (Fig. 2).

**Fig. 3:**
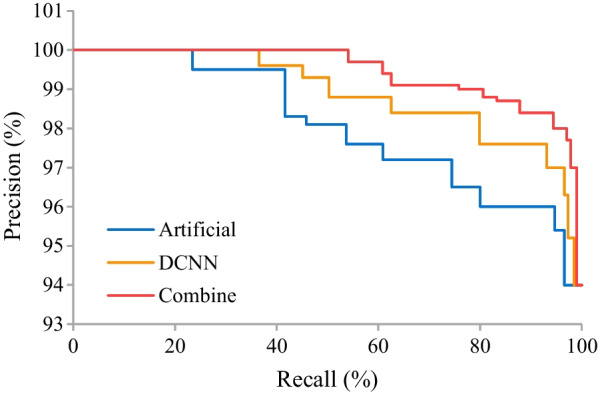
PRCs of computer CT intelligent detection

Precision-recall curves (PRCs) were drawn to show the effectiveness of the detection of OVCFs by artificial detection, DCNN model, and combined testing. It was demonstrated that the area under the curve (AUC) of artificial detection was the smallest, while that of combined testing was the largest (Fig. 3).

**Fig. 4:**
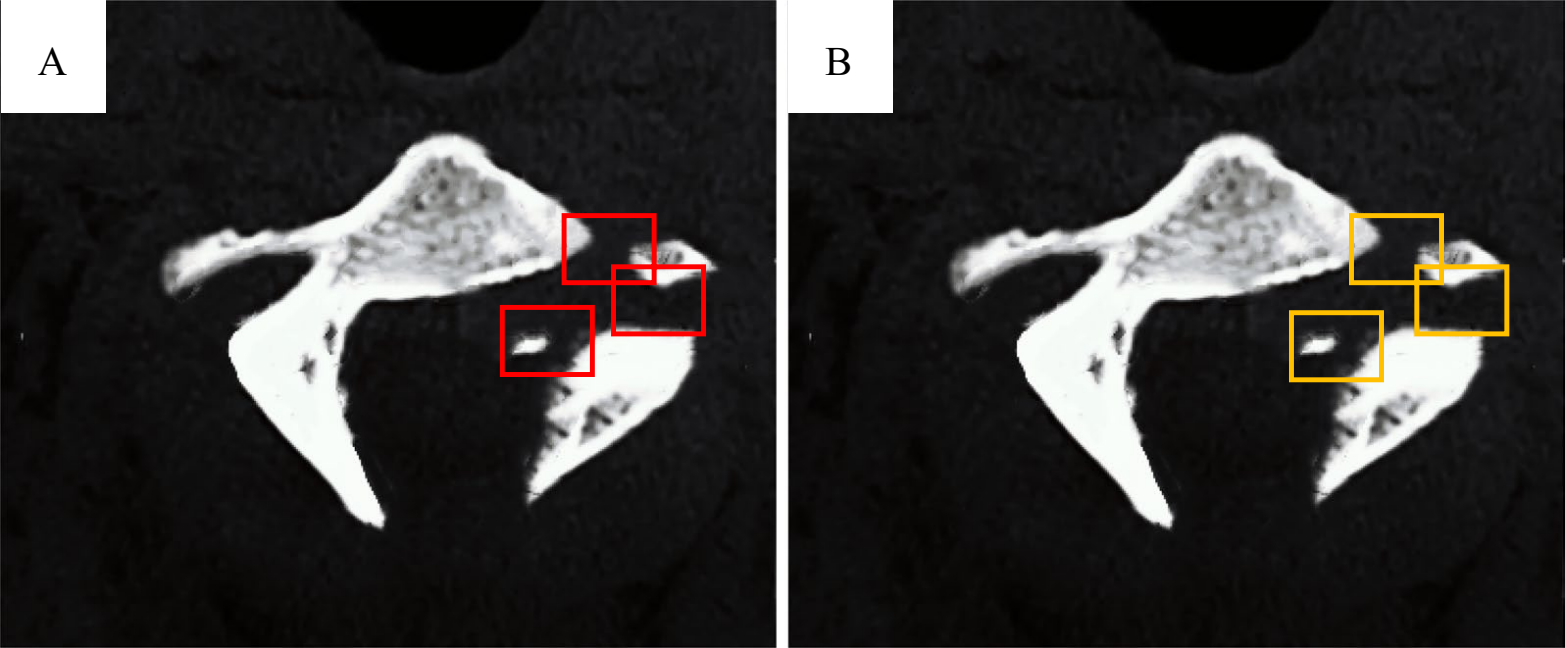
Effects of computer CT intelligent detection. **A** Artificial annotation of fractured sites in CT images. **B** Fractured sites in CT images annotated by DCNN model

The evaluation of the effects of the detection of fracture sites in CT images of OVCFs patients by artificial testing and DCNN model was compared. It was revealed that the above two detection methods had the same marking effects on fractured sites (Fig. 4).

### Comparison of ODI scores before and after treatment

**Fig. 5:**
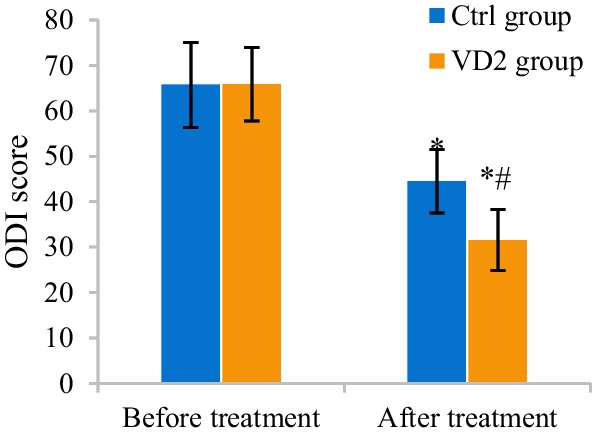
Comparison of ODI scores for the two groups. The comparison with ODI score for the same group before treatment revealed *P < 0.05. The comparison with ODI score for Ctrl group revealed #*P* < 0.05

The differences in ODI scores for Ctrl and VD2 groups before and after treatment were analyzed and compared. ODI scores for Ctrl and VD2 groups before and after treatment amounted to 65.72 ± 9.38 versus 65.83 ± 8.11 and 44.51 ± 7.02 versus 31.57 ± 6.73, respectively. After treatment, ODI scores for the two groups both remarkably declined (*P* < 0.05). Versus that for Ctrl group, ODI score for VD2 group also notably decreased after treatment (*P* < 0.05) (Fig. 5).

### Spinal ultrasound and CT imaging evaluation before and after treatment

**Fig. 6:**
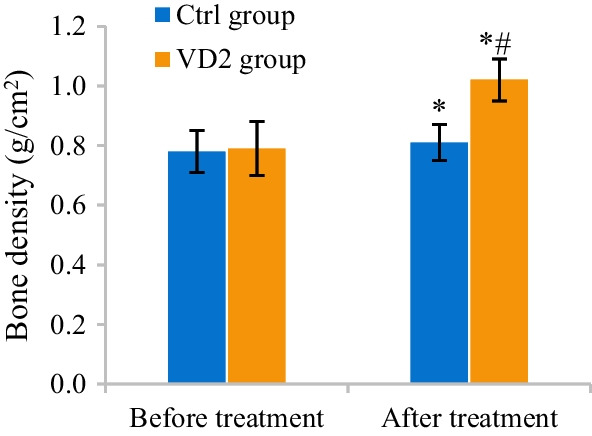
Comparison of bone density between two groups detected by quantitative CT. The comparison with bone density of the same group before treatment revealed *P < 0.05. The comparison with bone density of Ctrl group revealed #*P* < 0.05

Quantitative CT images were utilized to evaluate the difference in bone density among patients in Ctrl and VD2 groups before and after treatment. Bone density among patients in Ctrl and VD2 groups before and after treatment amounted to 0.78 ± 0.07 versus 0.79 ± 0.09 and 0.81 ± 0.06 versus 1.02 ± 0.07, respectively. After treatment, bone density among patients in the two groups both apparently rose (*P* < 0.05). Versus that among patients in Ctrl group, bone density among patients in VD2 group also increased after treatment (*P* < 0.05) (Fig. 6).

**Fig. 7:**
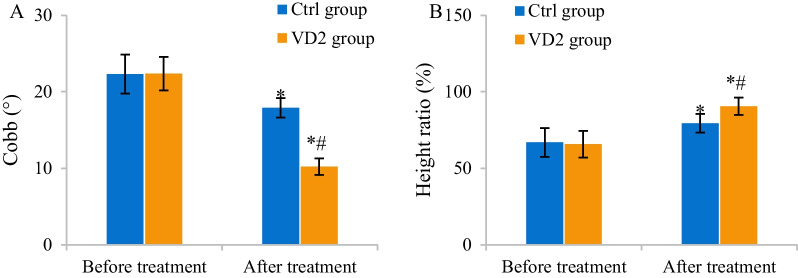
Comparison of Cobb angles and height ratio of injured vertebra between two groups. **A** Comparison of Cobb angles. **B** Comparison of height ratio of injured vertebra. The comparison with Cobb angles and height ratio of injured vertebra of the same group before treatment revealed *P < 0.05. The comparison with Cobb angles and height ratio of injured vertebra of Ctrl group revealed #*P* < 0.05

The differences in Cobb angles and the height ratios of injured vertebra among patients in Ctrl and VD2 groups before and after treatment were evaluated using quantitative CT and ultrasound images. Cobb angles of patients in two groups before and after treatment amounted to 22.31 ± 2.55° versus 22.36 ± 2.19° and 17.89 ± 1.27° versus 10.22 ± 1.08°, respectively. The height ratios of injured vertebra among patients in two groups before and after treatment amounted to 66.89 ± 9.43% versus 65.71 ± 8.77% and 79.36 ± 6.14% and 90.52 ± 5.58%, respectively. After treatment, Cobb angles remarkably declined while the height ratios of injured vertebra notably increased among patients in two groups (*P* < 0.05). Versus those among patients in Ctrl group, Cobb angle dramatically declined while the height ratio of injured vertebra apparently increased among patients in VD2 group after treatment (*P* < 0.05) (Fig. 7).

### Comparison of B-ALP and 25-OH-VD expressions before and after treatment

**Fig. 8:**
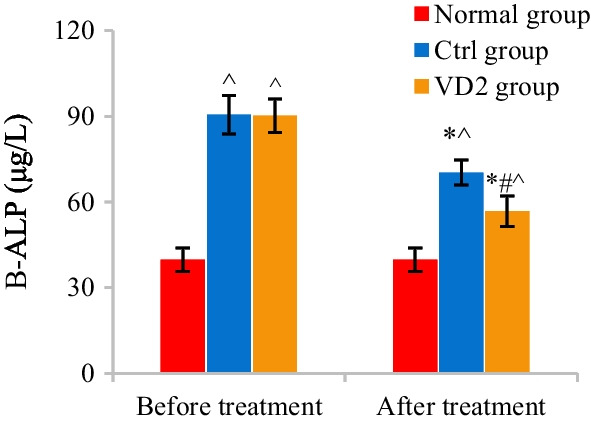
Comparison of B-ALP among patients in the three groups. The comparison with B-ALP of Normal group revealed ^*P* < 0.05. The comparison with B-ALP of the same group before treatment revealed *P < 0.05. The comparison with B-ALP of Ctrl group revealed #*P* < 0.05

The differences in B-ALP among patients in Normal, Ctrl, and VD2 groups before and after treatment were analyzed and compared. B-ALP among patients in the three groups before and after treatment amounted to 39.81 ± 4.14 μg/L versus 90.54 ± 6.71 μg/L versus 90.19 ± 5.89 μg/L and 39.81 ± 4.14 μg/L versus 70.28 ± 4.42 μg/L versus 56.70 ± 5.33 μg/L, respectively. Versus that of Normal group, B-ALP of Ctrl and VD2 groups both notably rose (*P* < 0.05). In contrast to that before treatment, B-ALP of Ctrl and VD2 groups both apparently declined after treatment (*P* < 0.05). Versus that of Ctrl group, B-ALP of VD2 group also remarkably decreased after treatment (*P* < 0.05) (Fig. 8).

**Fig. 9:**
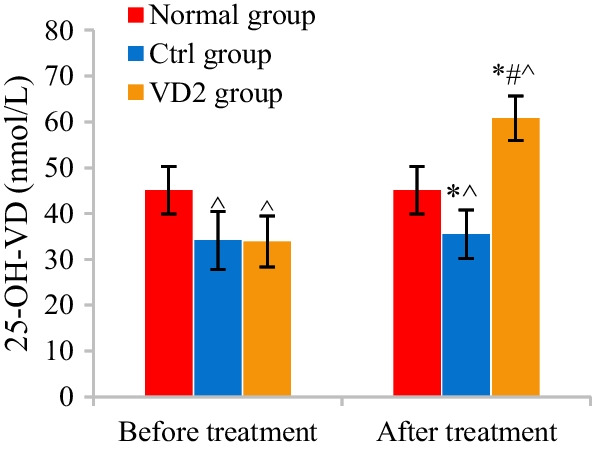
Comparison of 25-OH-VD among patients in three groups. The comparison with 25-OH-VD of Normal group revealed ^*P* < 0.05. The comparison with 25-OH-VD of the same group before treatment revealed *P < 0.05. The comparison with 25-OH-VD of Ctrl group revealed #*P* < 0.05

The differences in 25-OH-VD among patients in Normal, Ctrl, and VD2 groups before and after treatment were analyzed and compared. 25-OH-VD of Normal, Ctrl, and VD2 groups before and after treatment 45.06 ± 5.17 nmol/L versus 34.12 ± 6.32 nmol/L versus 33.87 ± 5.56 nmol/L and 45.06 ± 5.17 nmol/L versus 35.51 ± 5.29 nmol/L versus 60.83 ± 4.87 nmol/L, respectively. In contrast to that of Normal group, 25-OH-VD of Ctrl and VD2 groups both remarkably reduced (*P* < 0.05). Versus that before treatment, 25-OH-VD of VD2 group notably declined after treatment (*P* < 0.05), while that of Ctrl group showed no apparent changes (*P* > 0.05). Versus that of Ctrl group, 25-OH-VD of VD2 group remarkably increased after treatment (*P* < 0.05) (Fig. 9).

### Comparison of QOL scores after treatment

**Fig. 10:**
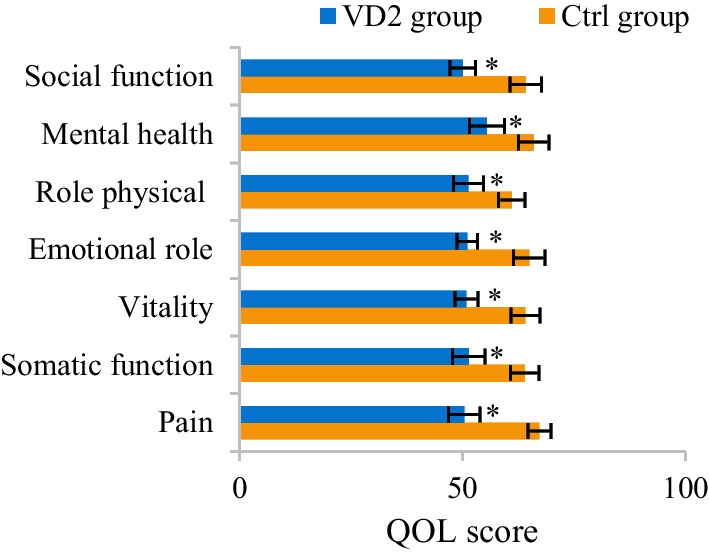
Comparison of QOL scores for patients in two groups. The comparison with QOL scores for the same group before treatment revealed *P < 0.05. The comparison with QOL scores for Ctrl group revealed #*P* < 0.05

The difference in QOL among patients in Ctrl and VD2 groups was analyzed and compared. The scores for pain, somatic function, vitality, emotional role, physical role, mental health, and social function among patients in Ctrl group amounted to 67.25 ± 2.59, 63.98 ± 3.17, 64.11 ± 3.26, 64.93 ± 3.54, 61.05 ± 2.97, 65.98 ± 3.41, and 64.17 ± 3.55, respectively. In contrast, the above scores for patients in VD2 group amounted to 50.38 ± 3.52, 51.41 ± 3.65, 50.89 ± 2.54, 51.05 ± 2.26, 51.32 ± 3.38, 55.47 ± 3.91, and 50.09 ± 2.85, respectively. Versus those for Ctrl group, all QOL scores for VD2 group notably increased after treatment (*P* < 0.05) (Fig. 10).

### Diagnostic values of B-ALP and 25-OH-VD for OVCFs

**Fig. 11:**
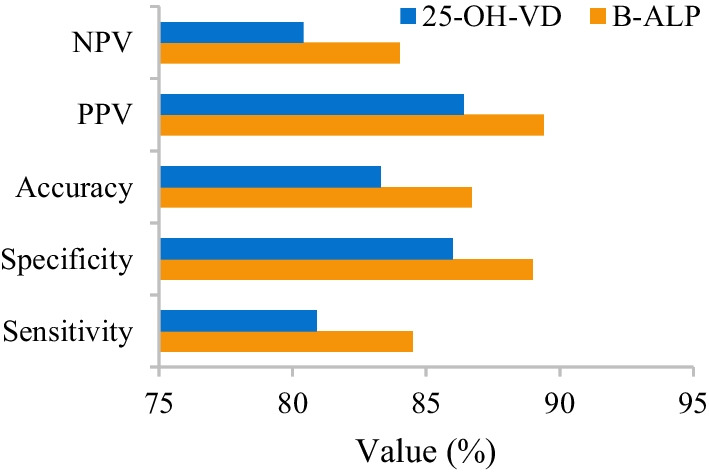
Comparison of the values of B-ALP and 25-OH-VD in the diagnosis of OVCFs

The differences in the sensitivity, specificity, accuracy, negative predictive values, and positive predictive values of B-ALP and 25-OH-VD for the diagnosis of OVCFs were analyzed and compared. The sensitivity, specificity, accuracy, negative predictive values, and positive predictive values of B-ALP and 25-OH-VD in the diagnosis of OVCFs amounted to 84.5%, 89.0%, 86.7%, 89.4%, and 84.0% and 80.9%, 86.0%, 83.3%, 86.4%, and 80.4%, respectively (Fig. 11).

**Fig. 12:**
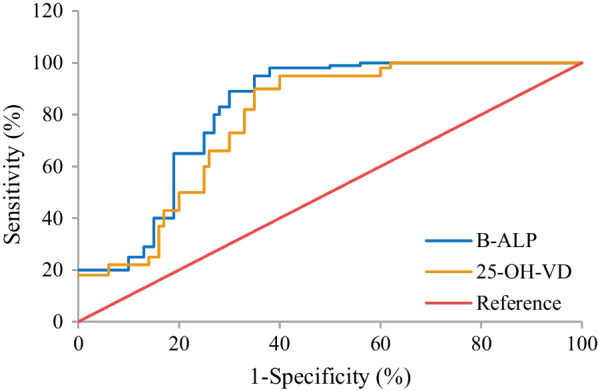
The values of the prediction and diagnosis of OVCFs by B-ALP and 25-OH-VD

ROCs were drawn to show the diagnostic values of B-ALP and 25-OH-VD for OVCFs. It was found that the critical values of the diagnosis of OVCFs by B-ALP and 25-OH-VD were 87.8 μg/L and 30.3 nmol/L, respectively, and AUC were 0.86 and 0.82 (Fig. 12).

### Correlation between B-ALP as well as 25-OH-VD and bone density

**Fig. 13:**
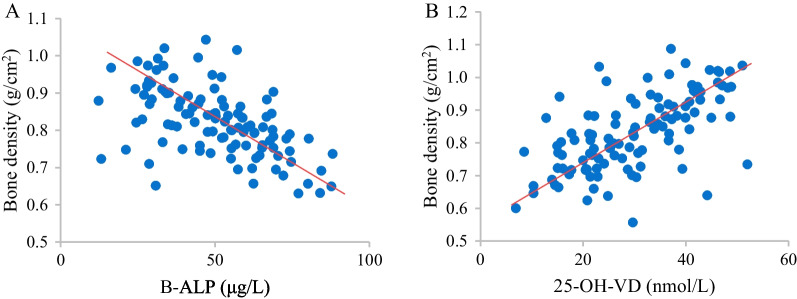
Analysis of the correlation between B-ALP as well as 25-OH-VD and bone density. **A** Correlation between B-ALP and bone density. **B** Correlation between 25-OH-VD and bone density

The correlation between B-ALP as well as 25-OH-VD and bone density was analyzed and compared. B-ALP was notably negatively correlated with bone density (*r* =  − 0.602, *P* < 0.05). 25-OH-VD was remarkably positively correlated with bone density (*r* = 0.576, *P* < 0.05) (Fig. 13).

## Discussion

With the recent increase of the incidence of spinal fracture caused by osteopenia and osteoporosis year by year, the main clinical symptoms include waist/back pain and spinal malformation, which seriously affect QOL [16]. With the progress of the disease, conservative drug therapy can’t effectively alleviate clinical symptoms, but aggravates the severity of osteoporosis and induce deep vein thrombosis and other complications [17]. Therefore, early diagnosis of OVCFs is of great significance for the improvement of prognosis. In the context of safe medical images, imaging examination is usually performed to assess the morphological changes of vertebral body and the status of fracture line for the clinical diagnosis of OVCFs [18]. Slightly deformed vertebral bodies can be detected by CT images. However, bone marrow edema state can’t be effectively detected [19]. To achieve computer automated detection effectiveness of CT images, DCNN model was selected for the classification and recognition of fractured regions in spinal CT images. It was demonstrated that precision, recall, and F1 of DCNN model for fracture recognition were higher than those of artificial annotation. What’s more, fractured sites could be localized by DCNN model. The above research findings revealed that computer intelligent technology could be utilized to classify and recognize fracture CT images for the automatic annotation of fractured sites, the reduction of workload of artificial annotation, the improvement of diagnostic rate, and the reduction of false negative and positive rates [20].

PVP is a main treatment method for OVCFs in clinical practice. Bone cement is injected to increase the stability of the injured vertebra and further promote the rehabilitation of spinal function as well as relieve clinical symptoms [21]. Vitamin D deficiency is common among patients with OVCFs. As a result, calcium absorption capacity is reduced, which causes fragility fracture or refractory fracture [22]. B-ALP is mainly concentrated in ossification sites of body and the specific marker for osteogenesis [23]. The relevant research findings demonstrated that B-ALP increased while 25-OH-VD declined among patients with OVCFs versus those among healthy population. An et al. [24] investigated the correlation between 25-OH-VD expression and bone density among male patients with diabetes and found that 25-OH-VD apparently declined among patients with diabetes and osteoporosis. Besides, 25-OH-VD was negatively correlated with bone density and B-ALP. It was suggested that the accuracy, sensitivity, and specificity of B-ALP and 25-OH-VD for the prediction and diagnosis of OVCFs were higher than 80.0%, 80.0%, and 85.0%, respectively. Anagnostis et al. [25] explored the correlation between bone turnover markers and bone mineral density (BMD) among male patients with hemophilia and found that B-ALP rose among patients with low BMD and B-ALP was negatively correlated with the history of fracture. Yu et al. [26] analyzed the changes of the expression of bone metabolism marker among patients primary spontaneous pneumothorax and found that 25-OH-VD was the risk factor for primary spontaneous pneumothorax and it was linearly associated with osteogenesis markers. The above research outcome was similar to the research finding that bone density was negatively correlated with B-ALP while positively correlated with 25-OH-VD. To sum up, B-ALP and 25-OH-VD could be adopted as the adjuvant biomarkers for the diagnosis of osteoporosis and the prediction of spinal fracture.

Vitamin D is lipid-soluble and it is an essential hormone that regulates bone metabolism. What’s more, it plays a vital role in promoting calcium absorption and regulating the stability of minerals and skeletal health [27]. Weaver et al. [28] assessed the risk of the prevention of fracture through calcium and vitamin D supplementation for adults among 30,970 included subjects. Fracture occurred among 2426 patients. The total risk of fracture prevention through calcium and vitamin D supplement decreased by 15% (95% confidence interval (CI) ranged between 0.73 and 0.95) and the risk of hip fracture reduced by 30% (95%CI ranged between 0.56 and 0.87). Schlewitz et al. [29] showed that osteoporosis occurred in vertebra and BMD apparently declined among the rats undergoing ovariectomy with D2 and D3 deficiency, which revealed that vitamin D supplementation was vital for fracture prevention. The impacts of postoperative intramuscular injection of vitamin D2 on bone density, spinal function rehabilitation, and QOL among patients with OVCFs. It was revealed that postoperative vitamin D2 supplementation could apparently improve 25-OH-VD and bone density. After the surgery, spinal ODI score declined, while various QOL scores rose. The above research findings were similar to the result obtained by Eskandarynasab et al. [30]. They found that bone mechanical strength of rats was enhanced, B-ALP was reduced, and osteoclasts were inhibited by applying phosphatidylserine nano-loaded vitamin D3 to the treatment of osteoporosis. Vitamin D could act on osteoblasts, osteoclasts, and bone mineralization. Hence, it could be involved in the process of union of fracture [31]. Based on the research findings, it was demonstrated that vitamin D combined surgery could notably promote the rehabilitation of spinal function and improve postoperative QOL.

## Conclusions

In the context of safe medical images, computer intelligent image analysis technique could be employed to apparently improve the efficiency of the classification and recognition of fractured sites in CT images. The result of fracture site localization was consistent with that of artificial annotation. B-ALP and 25-OH-VD was remarkably correlated with bone density and could be utilized for the diagnosis of osteoporosis and the prediction of spinal fracture. In addition, vitamin D was supplemented for OVCFs patients after PVP to effectively improve clinical symptoms, promote the rehabilitation of spinal function, and improve QOL. In this research, only the classification and recognition effects of computer intelligent technology on fractured sites in CT images were analyzed without the evaluation of the diagnostic effectiveness of B-ALP and 25-OH-VD for OVCFs. Besides, long-term therapeutic effects were not evaluated. This research was conducted to provide experimental data for the selection of early diagnostic markers for OVCFs and the promotion and application of adjuvant vitamin D therapy.

## Data Availability

The data that support the findings of this study are available on request from the corresponding author.

## References

[1] Li YY, Cui Y, Wang HL (2019). Effect of total flavonoids of Herba Taxilli on osteoporotic rats induced by retinoic acid. World J Tradit Chin Med.

[2] Hollensteiner M, Sandriesser S, Bliven E (2019). Biomechanics of osteoporotic fracture fixation. Curr Osteoporos Rep.

[3] Li HM, Zhang RJ, Gao H (2018). New vertebral fractures after osteoporotic vertebral compression fracture between balloon kyphoplasty and nonsurgical treatment PRISMA. Medicine.

[4] Prost S, Pesenti S, Fuentes S (2021). Treatment of osteoporotic vertebral fractures. Orthop Traumatol Surg Res: OTSR.

[5] Lv M, Zhou Z, Tang Q (2020). Differentiation of usual vertebral compression fractures using CT histogram analysis as quantitative biomarkers: a proof-of-principle study. Eur J Radiol.

[6] Foti G, Lombardo F, Guerriero M (2022). Management of vertebral compression fractures: the role of dual-energy CT in clinical practice. Radiol Med (Torino).

[7] Wan Z, Dong Y, Yu Z (2021). Semi-supervised support vector machine for digital twins based brain image fusion. Front Neurosci.

[8] Zhou X, Li Y, Liang W (2021). CNN-RNN based intelligent recommendation for online medical pre-diagnosis support. IEEE/ACM Trans Comput Biol Bioinf.

[9] Xie S, Yu Z, Lv Z (2021). Multi-disease prediction based on deep learning: a survey. CMES-Comput Model Eng Sci.

[10] Redlich K, Ziegler S, Kiener HP (2000). Bone mineral density and biochemical parameters of bone metabolism in female patients with systemic lupus erythematosus. Ann Rheum Dis.

[11] Zhang G, Zhu J, Zhang Q (2022). Effects of high viscosity bone cement percutaneous vertebroplasty on pain, bone specific alkaline phosphatase, Type-I collage cross-linked-telopeptide and Boneglaprotein levels in patients with osteoporotic vertebral compression fractures. Pak J Med Sci.

[12] Çolak Y, Afzal S, Nordestgaard BG (2020). 25-Hydroxyvitamin D and risk of osteoporotic fractures: mendelian randomization analysis in 2 large population-based cohorts. Clin Chem.

[13] Silva BC, Camargos BM, Fujii JB (2008). Prevalence of vitamin D deficiency and its correlation with PTH, biochemical bone turnover markers and bone mineral density, among patients from ambulatories. Arq Bras Endocrinol Metabol.

[14] Chen J, Xu C, Yu J. Vesselplasty for the treatment of osteoporotic vertebral compression fractures with peripheral wall damage: a retrospective study. Br J Neurosurg. 2020;1–11. 10.1080/02688697.2020.1862054 33319625

[15] Cho MJ, Moon SH, Lee JH (2021). Association between osteoporotic vertebral compression fractures and age, bone mineral density, and European quality of life-5 dimensions in Korean postmenopausal women: a nationwide cross-sectional observational study. Clin Orthop Surg.

[16] Vendeuvre T, Brossard P, Pic JB (2021). Vertebral balloon kyphoplasty versus vertebral body stenting in non-osteoporotic vertebral compression fractures at the thoracolumbar junction: a comparative radiological study and finite element analysis (BONEXP study). Eur Spine J: Off Publ Eur Spine Soc Eur Spinal Deform Soc Eur Sect Cerv Spine Res Soc.

[17] Kutsal FY, Ergin Ergani GO (2021). Vertebral compression fractures: still an unpredictable aspect of osteoporosis. Turk J Med Sci.

[18] Nakazawa F (2021). Subcutaneous edema on back detected by MRI in hospitalized patients with osteoporotic vertebral compression fracture. J Orthop.

[19] Zhao Y, Zhang T, Tang P (2022). Diagnostic value of SPECT/CT bone imaging in fresh osteoporotic vertebral compression fractures. Hell J Nucl Med.

[20] Aghnia Farda N, Lai JY, Wang JC (2021). Sanders classification of calcaneal fractures in CT images with deep learning and differential data augmentation techniques. Injury.

[21] Zhu J, Yang S, Cai K (2020). Bioactive poly (methyl methacrylate) bone cement for the treatment of osteoporotic vertebral compression fractures. Theranostics.

[22] Ko S, Jun C, Nam J (2021). Effects of vitamin D supplementation on the functional outcome in patients with osteoporotic vertebral compression fracture and vitamin D deficiency. J Orthop Surg Res.

[23] Tariq S, Tariq S, Shahzad M (2021). Association of serum chemerin with calcium, alkaline phosphatase and bone mineral density in postmenopausal females. Pak J Med Sci.

[24] An ZM, Huang MJ, Zhang M (2009). Relationship of 25(OH)VD with bone mass and other indicators in male patients with diabetes mellitus. J Sichuan Univ Med Sci Edit.

[25] Anagnostis P, Vakalopoulou S, Vyzantiadis TA (2014). The clinical utility of bone turnover markers in the evaluation of bone disease in patients with haemophilia A and B. Haemophilia: Off J World Fed Hemophilia.

[26] Yu L, Li H, Hou S (2016). Abnormal bone mineral density and bone turnover marker expression profiles in patients with primary spontaneous pneumothorax. J Thorac Dis.

[27] Fischer V, Haffner-Luntzer M, Amling M (2018). Calcium and vitamin D in bone fracture healing and post-traumatic bone turnover. Eur Cell Mater.

[28] Weaver CM, Alexander DD, Boushey CJ (2016). Calcium plus vitamin D supplementation and risk of fractures: an updated meta-analysis from the National Osteoporosis Foundation. Osteoporos Int: J establ Result Coop Eur Found Osteoporos Natl Osteoporos Found USA.

[29] Schlewitz G, Govindarajan P, Schliefke N (2013). Ovariectomy and calcium/vitamin D2/D3 deficient diet as a model of osteoporosis in the spine of Sprague-Dawley rats. Z Orthop Unfallchir.

[30] Eskandarynasab M, Etemad-Moghadam S, Alaeddini M (2020). Novel osteoprotective nanocochleate formulation: a dual combination therapy-codelivery system against glucocorticoid induced osteoporosis. Nanomed Nanotechnol Biol Med.

[31] Thanapluetiwong S, Chewcharat A, Takkavatakarn K (2020). Vitamin D supplement on prevention of fall and fracture: a meta-analysis of randomized controlled trials. Medicine.

